# Intentional Observer Effects on Quantum Randomness: A Bayesian Analysis Reveals Evidence Against Micro-Psychokinesis

**DOI:** 10.3389/fpsyg.2018.00379

**Published:** 2018-03-21

**Authors:** Markus A. Maier, Moritz C. Dechamps, Markus Pflitsch

**Affiliations:** Department of Psychology, Ludwig-Maximilians-Universität München, Munich, Germany

**Keywords:** quantum observation, micro-psychokinesis, random number generator, RNG, model of pragmatic information

## Abstract

Intentional effects of human observation on the output of quantum-based random number generators (tRNG) have been studied for decades now. This research has been known as micro-psychokinesis (micro-PK) and many studies in the field reported evidence for mentally induced non-random deviations from chance. A most recent meta-analysis from Bösch et al. (2006) revealed a very small and heterogeneous overall effect size that indicated a significant deviation from chance across studies. There remains doubt among the scientific community on the existence of micro-PK given: (i) the small and heterogenous effect; and (ii) the fact that several independent replication attempts of prominent studies failed to confirm the original results. The study presented here was intended to provide decisive evidence *for* or *against* the existence of micro-PK. An online experiment with 12,571 participants was conducted. The Bayesian analysis revealed strong evidence for H_0_ (BF_01_ = 10.07). Thus, micro-PK did not exist in the data. A closer inspection of the temporal change of the effect seemed to suggest a non-random oscillative structure with a higher frequency than observed in simulated data. The possible role of entropy and the relation to the model of pragmatic information from von Lucadou (2015) is discussed.

## Introduction

From time immemorial, humans have been fascinated by the relationship between the spiritual and physical worlds. Speculation focuses on both the mind and body/brain connection and the relationship between the mind and the outside physical world. Specifically, the idea that the mind can materialize ideas/desires or modify aspects of physical reality has been widely addressed in religion, mythology, and philosophy. Descartes, the great philosopher, mathematician, and devoted gambler, wrote about his personal experience with mind and reality during the 1640s. He observed that an otherwise random gambling outcome could be positively influenced by a gambler with a happy and optimistic mood (cited in Davidenko, 1990, p. 306). It was another 300 years before empirical examination of mind-matter interactions occurred. In the 1940s Rhine (1944) explored mind effects on dice tosses. Since then, there have been a large number of studies that examined mental influence or mentally induced statistical variations on inanimate probabilistic systems such as tumbling dice, tossing coins, or random number generators (RNGs). This research is now incorporated in work that is termed “micro-psychokinesis” (micro-PK) research (Varvoglis and Bancel, 2015).

The use of quantum-based RNGs, so-called true random number generators (tRNGs), as an optimal source of randomness has become the standard in micro-PK research (e.g., Jahn et al., 1980). In meta-analyses of 100s of studies performed using tRNGs, a small but significant effect of the human mind on non-random deviations from chance was found (Bösch et al., 2006; see also Radin and Nelson, 1989, 2003). Despite this, micro-PK is not a generally accepted phenomenon in science. This is because meta-analysis has several flaws, including the ability to be biased by the inclusion of successful studies. In addition, high-powered attempts to replicate positive micro-PK tests have not been successful (e.g., Jahn et al., 2000; Maier and Dechamps, in press). The goal of this study was to conduct a decisive scientific test for micro-PK. A large-scale assessment was performed that used Bayesian techniques to consolidate data until clear evidence *for* or *against* the existence of micro-PK was found.

Micro-PK research using tRNGs began in the 1960s with researchers using quantum states as a source of true randomness. Over the following decades, the body of research data increased (e.g., Schmidt, 1970; Jahn et al., 1980, 1987). A meta-analysis by Radin and Nelson (1989), including 597 studies conducted up until 1987, found a strong effect supporting micro-PK. This result was confirmed 15 years later in a meta-analysis with additional 176 new studies (Radin and Nelson, 2003). However, these meta-analyses included studies using both tRNGs and poorer-quality algorithmically-based RNGs. A more recent meta-analysis by Bösch et al. (2006) only included studies using tRNGs. This analysis of 380 studies undertaken between 1961 and 2004 identified a very small and heterogeneous effect that indicated a significant deviation from chance (Bösch et al., 2006). A significant negative correlation between sample size and effect size was also found (Bösch et al., 2006). Given the small, heterogeneous effect and this correlation, the authors concluded that the observed effect might have been caused by publication bias (Bösch et al., 2006); other researchers have questioned this interpretation (Radin et al., 2006) and a deeper inspection of the Radin and Nelson (1989, 2003) meta-analyses confirms that these aspects do not apply to their data. Nevertheless many scientists agree that evidence derived from meta-analyses alone does not provide a convincing argument for the existence of micro-PK effects. In addition, meta-analysis methods have recently been criticized, especially with regard to the impact of heterogeneity (e.g., Ioannidis, 2016). This has led to the suggestion that “*a single high-quality, well-reported study can be recommended instead of a statistical synthesis of heterogeneous studies*” (Brugha et al., 2012, p. 450). A similar suggestion was made by van Elk et al. (2015).

However, high-quality studies aimed at replicating existing results are scarce in micro-PK research. One example is the Jahn et al. (2000) study that utilized research teams from the PEARlab at Princeton University, the Grenzgebiete der Psychologie und Psychohygiene at Freiburg, and the Center for Behavioral Medicine at the Justus Liebig University Giessen. They attempted to replicate the Jahn et al. (1987) benchmark study involving 97 subjects and data from 2.5 million micro-PK trials. The attempted replication, with 227 participants and over 2 million trials, failed to confirm the original results (Jahn et al., 2000). Another is the Maier and Dechamps (in press) micro-PK research that reported on two micro-PK studies using Bayesian methods. The authors reported strong evidence supporting micro-PK in Study 1 (BF_10_ = 66.7). However, in Study 2, a pre-registered, high-quality replication of Study 1, they found strong evidence for the null effect (BF_01_ = 11.07). Failure of these high-powered studies to replicate earlier results also raised doubts about the existence of micro-PK. To date, robust and convincing evidence for micro-PK is missing. One potential factor explaining some of the replication failures might lie in the way intentional observation was manipulated. So far, to the greater part explicit goal manipulation procedures have been used in micro-PK research. To our view, this might be less fruitful than a more subtle implicit manipulation. We will outline this in the following paragraphs.

In parallel with empirical efforts, theoretical models have been proposed to explain and predict the effect of the human mind on quantum-based outcomes. Orthodox quantum physics regards the randomness that occurs during the act of measuring a quantum system as ontic and inherent in nature (see Greenstein and Zajonc, 2006). For example, the location of one electron is not classically defined before measurement but needs to be described as a superposition of several simultaneous locations. This phenomenon is captured by the mathematical concept of a “wave function” (Schrödinger, 1935). After measurement, an electron is found in one specific location with a probability given by the square of the amplitude of the wave function (Born, 1926). Thus, the results of a quantum measurement are only predictable with likelihoods, never with certainty. In addition, no measurement or observation can influence the probabilities to produce deviations from randomness. Although this is the standard interpretation of quantum mechanics, some revised versions of quantum theory have recently been developed that allow for mental processes during observation to slightly influence the likelihood of an outcome of a quantum process.

Atmanspacher et al. (2002) presented the Generalized Quantum Theory (GQT) (see also Römer, 2004; Filk and Römer, 2011; Atmanspacher and Filk, 2012). Here, a measurement in a quantum experiment is considered an observation when knowledge transfer takes place (epistemic split). This epistemic split occurs when unknown potential quantum alternatives are transferred into conscious knowledge. The knowledge transfer can be shaped by the observer’s mind set, for example, his or her intentions. Observer effects are thus described as entanglement correlations between the intentional observer and the observed system (von Lucadou and Römer, 2007). Consequently, non-random deviations from quantum probabilities are allowed. This theory also proposes that such deviations should decline shortly after their first detection. The reason for this is that deviations from randomness constitute a severe violation of the “no signal theorem” in quantum mechanics. To utilize this as evidence-based documentation is forbidden. This leads to the disappearance of the micro-PK effect in later replication attempts. Thus, on the macroscopic level, the no signal theorem is saved (von Lucadou, 2006, 2015); in other words, if a signal transfer occurs, it cannot be used intentionally because its appearance and disappearance vary unsystematically. Maier and Dechamps (in press) also suggested that a micro-PK effect changes over time and argued by referring to the entropy principle that such effects might behave like dampened harmonic oscillations, reflecting the interplay between the quantum-PK effect and its counter-mechanism ‘entropy.’

Another theory is the OrchOR model proposed by Penrose and Hameroff (2011; see also Penrose, 1989, 1994; Hameroff and Penrose, 1996; Hameroff, 2012). Similar to Atmanspacher et al. (2002), they view the act of measurement as a transfer from unconscious knowledge about the nature of a quantum state into a conscious experience of its exact existence. This transfer occurs through quantum gravitation and can be affected by Platonic values directly related to gravity. These values amongst others include mental concepts (Hameroff and Chopra, 2012). Thus, intentional observers might be able to non-randomly influence the transition of potential quantum states into one specific classical state. Both approaches have in common that the mental effect on quantum randomness takes place before the transition from the unconscious quantum to the conscious classical state. Micro-PK should thus be mainly affected by unconscious-related states of mind of the observer (Maier and Dechamps, in press).

Given this premise, in our study we put participants who observed the outcomes of a quantum experiment in a mindset that was related to specific unconscious inner states. Participants were presented with a brief relaxation and optimism inducing meditative episode before they participated in the experiment. This was designed to stimulate an inner, deeply rooted belief that everything was good and will remain good. On an unconscious level, this should attract more positive outcomes than negative ones during randomly chosen stimulus presentations in a micro-PK experiment with a tRNG. The stimuli selected by a quantum based RNG at each trial consisted of positive and negative pictures and auditory stimuli. Overall, we expected that the average mean score for positive stimuli presentations should be above chance (50%). No decline or harmonic oscillation effects were expected at the beginning of the study and were thus not the focus of our predictions. They are explored in the additional analyses of the result section. The main goal of our research was to provide a decisive, high-quality test for micro-PK. We therefore decided to apply a Bayesian testing approach. This method allows for data accumulation until a stopping criterion (i.e., a pre-specified amount of evidence) has been reached. As effects expected from micro-PK would be small, it was clear we would need a sample size in the 1000s.

## Materials and Methods

Participant recruitment and data collection were organized by Norstat, Germany^1^, a professional data collection company specializing in online polling and testing with access to 650,000 potential participants in 18 European countries. The participant pool consists of pre-profiled volunteer adults and undergoes constant quality control.

### Participants

We decided to work with participant pools from three countries, resulting in a sample highly representative of the European population (see **Table 1**).

**Table 1:** Overview of the sample pools in Norstat.

Country	Pool size	Gender distribution (in %) female/male	Age distribution (in %) 18–24/25–34/35–44/45–54/55+
Germany	110,000	60/40	19/23/20/20/18
Spain	10,500	58/42	10/21/32/24/13
Italy	50,000	64/36	12/25/28/21/14


An invitation to participate in the study was sent from Norstat to a random selection of participants daily, aiming for a completion rate of about 100 per day. About 20–25% of invited individuals completed the study.

### Ethics Considerations

The experiment was approved by the ethics boards of Norstat and the Department of Psychology (LMU). Norstat obtained written consent from participants electronically by having them press an “accept” button^2^. Within the consent form, participants were informed in general terms about the study and advised that participation was voluntary. Participants could also withdraw at any point during the study. All data were coded, stored, and analyzed anonymously.

### Data Collection

The final sample size was not predefined. Instead an accumulative data collection and analysis strategy using Bayesian inference techniques for hypotheses testing was used (see Wagenmakers et al., 2011). This approach allows for data accumulation (i.e., additional subjects can be tested and results added into the data set) until a specified Bayes factor (BF) for H_1_ (or H_0_) has been reached. It also allows an option to stop data collection at a predetermined BF, so it is a more effective way of hypothesis testing than frequentist inference methods. We used BF = 10 as a stopping point for evidence collection of both an effect and a null effect.

The Bayesian approach provides information on how to update our beliefs given new incoming data. Bayesian methods accumulate data concerning the effect in question and repeatedly update the likelihood for an effect given additional data. The strength of evidence for the effect is considered dependent on both the likelihood of the data given that H_0_ is true as well as the likelihood of the data given H_1_ is true. Those two likelihoods are pit against each other leading to the so-called BF. The BF describes the relative amount of evidence that the data provide for or against a postulated effect. In this way, the existence (H_1_) and the non-existence (H_0_) of an effect can be tested. A BF of 10 or higher is considered to indicate strong evidence for H_1_ or H_0_, respectively. For instance, a BF_10_ = 10 means that the H_1_ is 10-times more likely to be true than the H_0_.

To calculate the BF, a probability distribution for effect size that is centered around zero with scale parameter r needs to be specified *a priori*. This Cauchy distribution (δ ∼ Cauchy [0, r]) identifies the prior, i.e., the likelihood of the data given there is an effect, p(data|H_1_). Wagenmakers et al. (2011) recommend an r equal to 1. The statistic software JASP designed to perform basic Bayesian analyses uses a default r of 0.707. Other authors recommend a lower r of 0.5 (Bem et al., 2011) or of 0.1 (Maier et al., 2014) knowing that PSI effect sizes are usually very small (i.e., mostly in the range of 0.1 to 0.2). The choice of the prior provides a degree of freedom within the Bayesian approach. For data analysis in the studies presented here we decided to use a r of 0.1, i.e., δ ∼ Cauchy (0, 0.1). This parameter was selected before data collection had been started.

We also decided in advance to analyze the data with a Bayesian one sample *t*-test using a one-tailed approach given our directed prediction. On a regular basis, almost every week, a one-sample *t*-test was performed testing the actual sample’s mean score of positive stimuli presentations against chance (50%). For all Bayesian analyses, the statistical software tool JASP (Version 0.8.2, JASP Team, 2017) was used. This was repeated over several months from November 2016 to July 2017 until the stopping criterion was met.

#### Final Sample

When the criterion to stop was satisfied (BF > 10), a total of 12,571 participants had been tested from three different countries. Due to a technical difficulty and some participants quitting the survey immediately after completing the study, demographic data was only available for 11,158 of the participants. Mean age of the final sample was 48.73 (*SD* = 13.60; range from 16 to 90) with 5,617 females (50.3%) and 5,541 males (49.7%). **Table 2** provides more demographic details of the participant sample.

**Table 2:** Overview of the participant sample.

Country	Participants	Demographic data	Female/male (in %)	Mean age
Germany	10,316	9,015	51.0/49.0	48.78 (*SD* = 13.76)
Spain	1,130	1,116	47.7/52.3	46.9 (*SD* = 10.78)
Italy	1,125	1,027	47.8/52.2	50.22 (*SD* = 14.67)
Total	12,571	11,158	50.3/49.7	48.73 (*SD* = 13.60)


### Materials

A survey was created that included a link to the study materials on our webserver.

#### Experimental Program

The study was constructed as an online experiment. This means participants were not tested in a laboratory but could participate from any computer with internet access and audio output. The experiment was displayed in the computer’s browser in full-screen mode. It was implemented with jsPsych (de Leeuw, 2015^3^), a JavasScript library for creating and running behavioral experiments in a web browser and ran on a dedicated webserver in the university’s computer center. The Quantis tRNG, used as random source for stimulus selection, was located in the same room and connected to the designated server via USB.

#### Stimuli

Visual stimulus material was obtained from Shutterstock^4^, a provider of royalty-free stock photos. Out of the library of around 125 million photographs, 100 pictures reflecting a positive prevailing mood and 100 pictures reflecting a negative one were selected. Positive picture material consisted of photos showing aspects of social belonging and affiliation, landscape shots, and pictures of cute animals. The negative material was selected to evoke displeasure within the participants; this was accomplished through pictures depicting imminent danger (e.g., attacking predators or weapons directed at the viewer, imagery provoking distress or misery, or pitiful and nauseating images). Picture selection was performed by the first and the second author based on their experience in emotion induction.

In order to intensify the mediated affect, a multisensory approach was used and audio stimuli were presented in addition to the images. The positive and negative impacts were conveyed by consonant and dissonant chords respectively. These piano chords consisted of tones that either harmonize well (i.e., produce a harmonious and melodious experience) or tones that harmonize poorly (i.e., form sharp dissonances, which are usually perceived as unpleasant). A total of eight consonant and eight dissonant chords were generated out of which two positive and two negative ones were selected by the experimenters for the study.

#### Generation of Quantum Randomness

For the purpose of random number generation, a quantum number generator (Quantis-v10.10.08) developed by the company idquantique from Geneva, Switzerland was used on the webserver^5^. This apparatus produces quantum states by using photons that are sent through a semi-conductive mirror-like prism. Each photon has an equal chance of being deflected in one of two directions, producing a superposition of both states, until a measurement is performed. Upon measurement, the photon is found on either route with 50% probability which is then transformed into a numerical score such as 0 or 1, depending on the track it was found (technically Quantis transforms 8 such bits into 1 Byte). This procedure is thus a reenactment of the famous double-slit experiment known in quantum physics testing the wave-particle duality. The hardware passed validation tests of randomness, such as the DIEHARD and the NIST tests (see certificates from various independent agencies on the website), and is regarded as one of the most effective tRNG worldwide (Turiel, 2007). The tRNG was connected to the server via USB. Since it operates without a buffer, it was ensured that the bit responsible for the selection of the stimuli was created directly before the presentation. A user monitoring code made sure that different participants did not access the tRNG at the exact same time but that everybody receives an individual bit.

### Procedure

Participants received an email from the data collection company inviting them to take part in a survey. They were asked to relocate, if necessary, to an undisturbed environment. The participants’ audio was tested by playing a short audio clip and asking them for its content. If they answered correctly, they were forwarded to the university’s webserver, where the experiment was displayed in full-screen mode. Participants were asked to close their eyes and listen to a pre-recorded relaxation exercise designed to put them in a relaxed and optimistic mood. The exercise was repeated once with a total playing time of about 2 min^6^. It was available in German, Italian, and Spanish and spoken by a native speaker. The text of the relaxation exercise was:

Leave all your thoughts and worries behind you. Breath slowly and calmly. Focus only on your breathing. Slowly and calmly… Slowly and calmly… slowly and calmly… slowly and calmly. You are feeling completely peaceful and relaxed and fully in the present. Release all your tension. Relax your muscles. You are feeling comfortable and safe! Completely comfortable and safe.

Following the relaxation exercise, participants were advised about the study. They were told that they would be presented with pleasant and unpleasant images and sounds and that they could abort the experiment at any time by closing the window. Presentation of stimuli began after these instructions.

**FIGURE 1:**
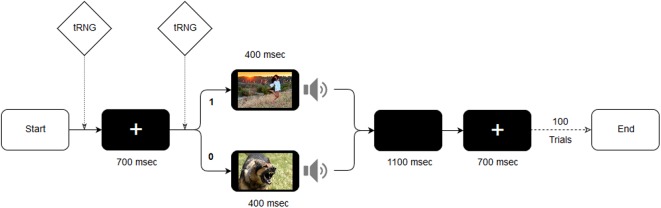
Stimulus selections and presentation times during one trial.

During each trial, the tRNG chose a random number between 1 and 100 to decide which visual and auditory stimuli would be displayed, then a random number between 0 and 1 to determine whether the stimuli would be positive or negative. During this process, a fixation cross was shown to the participant for 700 ms. The stimuli (picture and sound) were presented for 400 ms. Before the next trial, a black screen was shown for 1100 ms. This process is illustrated in **Figure 1**. A total of 100 trials were performed on each participant, which took approximately 6 mins.

After completing the task, participants were asked to fill out a short questionnaire. With regards to our relaxation induction, we asked participants to indicate their belief toward a general contentedness and hopeful confidence by asking them to rate the following statement: “I am strongly convinced that everything is going to be fine” on a seven-point scale from *Not true* to *Very True*.

Subsequently stimulus seeking was assessed with a scale constructed by Bem et al. (2011) that contained two statements: “I am easily bored” and “I often enjoy seeing movies I’ve seen before” (reverse scored). Responses were recorded on five-point scales that ranged from *Very Untrue* to *Very True* and averaged into a single score ranging from 1 to 5 (Cronbach’s α = 0.59).

Furthermore, we constructed a self-efficacy attitude measure related to general life outcome expectancies. This scale comprised the following six statements: “In life, you don’t get anything for free,” “You have to fight for everything,” “Life generally doesn’t mean well for me,” “You have to take stick a lot if you want to succeed,” “Nothing is going to change,” and “When it rains, it pours.” Responses were recorded on a seven-point scale from *Not true* to *Very true* and averaged into a single score. The scale provides a good reliability (Cronbach’s α = 0.80).

Lastly we asked participants to fill out the Life Orientation Test-Revised (LOT-R; Scheier et al., 1994). This questionnaire assesses generalized optimism (Cronbach’s α = 0.76) and pessimism (Cronbach’s α = 0.73) with three items each.

## Results

To explore the effectiveness of our relaxation manipulation, we first analyzed the single item measure “I am strongly convinced that everything is going to be fine.” Our hypothesis was that the sample’s mean score of this conviction was above the average. A one sample *t*-test (two-tailed) testing the item’s mean score against 4, which was the exact midpoint of a seven-point scale (ranging from not at all to very much), yielded a significant effect, *t*(11157) = 67.05; *p* < 0.001. On average the mean rating was significantly above the midpoint of the scale, *M* = 4.96 (*SD* = 1.51).

Next the observer effects on picture selection will be reported. The data were analyzed on average every week by the experimenter, the second author, depending on the number of participants tested during the preceding days (see Bayesian approach described above).

**FIGURE 2:**
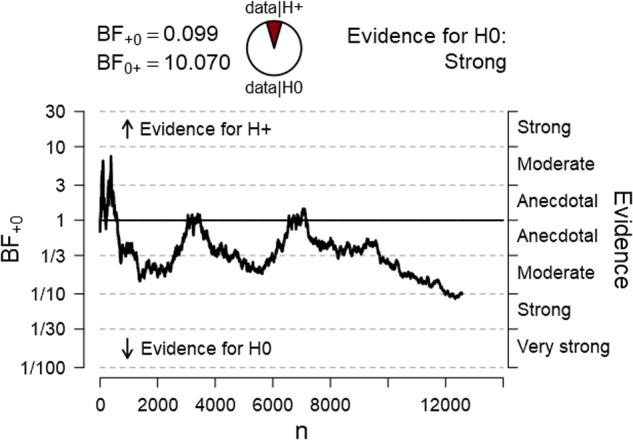
Sequential Bayesian one-sample *t*-test analysis of the percentage of positive pictures across all 12,529 participants.

The study tested the hypothesis that after being exposed to a relaxing and optimism inducing intervention, participants will, on average, observe more positive stimuli than expected by chance. The dependent variable was the percentage of positive stimuli achieved by each participant across 100 trials. The average percentage of positive stimuli for all participants was then tested against the 50% score. The final Bayesian one sample *t*-test (one-tailed) with 12,571 participants revealed a BF_01_ of 10.07 for H_0_. The mean score for positive stimuli for all participants was *M* = 50.02%, *SD* = 5.06, providing very strong evidence for a null effect, with no deviation from chance. **Figure 2** represents a sequential analysis of the BF across all participants in the order of testing.

**FIGURE 3:**
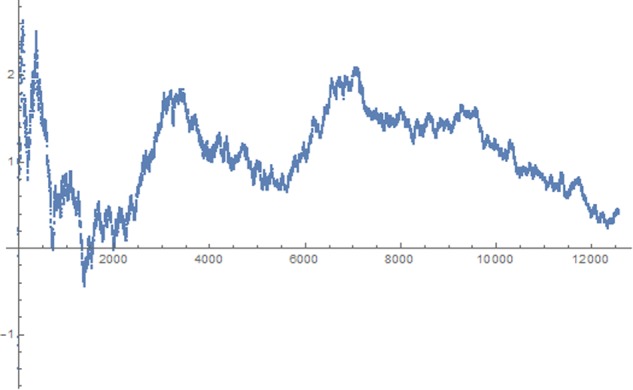
Sequential analysis of data by computing a cumulative z-score from the percentage of positive stimuli obtained after each participant.

A standard practice in micro-PK research is to display the effect and its change over the time of data collection as a cumulative z-score. This data sequence is shown in **Figure 3**.

As depicted in the graph, the effect went in the predicted direction almost entirely throughout the experiment and several times hit the 1.96 z-score line but then finally dropped to zero. An identical variation in effect can be seen in the BF sequential analysis.

In an additional set of analyses we also explored the relationship between the personality variables assessed from 11158 participants and the mean number of positive stimuli obtained from each individual. No significant correlations between number of positive stimuli and general life outcome expectancies (GLOE6), generalized optimism (LOT_Opt) and pessimism (LOT_Pess) or Stimulus Seeking was found (see **Table 3**).

**Table 3:** Bayesian Pearson correlations between number of positive stimuli and personality variables.

		Pos_Imgs	GLOE6	LOT_Opt	LOT_Pess
Pos_Imgs	Pearson’s r	—			
	BF_10_	—			
GLOE6	Pearson’s r	-0.015	—		
	BF_10_	0.039	—		
LOT_Opt	Pearson’s r	-0.005	-0.301	—	
	BF_10_	0.014	1.682e+228	—	
LOT_Pess	Pearson’s r	-0.009	0.615	-0.425	—
	BF_10_	0.019	∞	∞	—
SS	Pearson’s r	0.002	0.025	-0.149	0.087
	BF_10_	0.012	0.340	3.299e+52	4.088e+16


## Discussion

The results of our study provide strong evidence for H_0_, indicating no deviation of the mean number of positive stimuli from chance in our sample. Relaxed and optimistically induced participants who passively observed the pictures and auditory stimuli, chosen at each trial by a highly sophisticated and effectively working quantum RNG, seemed not to unconsciously affect the quantum process toward non-randomness. The data support the null hypothesis that predicted no mental effects on quantum randomness. A BF higher than 10 also underlines the robustness of this effect. In sum, the evidence speaks against the revised quantum models of Atmanspacher et al. (2002) and Penrose and Hameroff (2011) which postulate non-random deviations of quantum outcomes by deeply rooted mental activity. The data rather support original quantum theoretical interpretations from Bohr, Bohm, Wigner, and von Neumann that claim the observer has no active influence on the probabilities of quantum experimental outcomes (see Greenstein and Zajonc, 2006; Byrne, 2010). Although there are many possibilities as to why an effect may not have occurred in this experiment (e.g., failure of the induction method, distractions on behalf of the subjects during performance, or low quality of stimuli used), we assume that these and other factors would only increase error variance. Even then, the power of several 1000 subjects should be sufficient to detect an effect given a high error variance. The study was designed to overcome these limitations with an enormous power and to provide a sincere test of the proposed micro-psychokinetic mind-matter interaction. We thus believe that the null effect documented here might very well-constitute a real absence of a mental influence on quantum randomness at least at the level of the average mean score for positive stimuli. This would fit with earlier skeptical arguments raised by Bösch et al. (2006) against micro-psychokinesis reported in meta-analyses who suggest that those effects might be due to publication biases. In addition, no moderating effects of personality traits were found.

## Additional Analysis

The sequential Bayesian analysis and the z-score accumulation across participants also allowed for a closer inspection of statistical trends in the data. Interestingly, there seems to be a pattern of repeated change. The micro-PK effect appears to be alternatingly increasing and decreasing several times during the data collection period. This fact is noteworthy, since a similar observation has recently been made in comparable micro-psychokinesis studies investigating uninstructed goal-dependent observation effects on tRNG’s outputs (Maier and Dechamps, in press). In this research, two studies have been performed testing the hypothesis that smokers would cause a substantial deviation from chance on the mean number of cigarette-related pictures being presented. Without going into substantial detail, the general outcome of the studies was that the effect increased to an overall BF of above 100 until the beginning of Study 2; subsequently, there was a reversion of the effect back to a BF of 1 at the end of Study 2. This appearance-disappearance-pattern was not present in the non-smokers data nor in a simulation where no observers were present. In the latter two cases, no effect was there at any time. In addition, other research groups who extensively studied micro-psychokinesis also report such a decline of the effect during replication attempts (Jahn et al., 2000). Reports of similar decline effects in other studies complete this picture (see Radin, 2006).

Given these unexpected, yet broadly manifesting appearance-disappearance-patterns, theoretical efforts have been undertaken to understand such decline effects in micro-PK. One model that tried to understand the nature of this empirical phenomenon was proposed by von Lucadou (2006, 2015). His theory refers to the idea of Pragmatic Information and applies it to observer-related quantum effects. According to this theory, the novelty of a finding is complementary related to its likelihood of confirmation (i.e., the more novel a quantum effect, the lower the likelihood of a successful replication). The supposed principle behind this mutual relation is that quantum effects, such as micro-PK, violate the “no-signal theorem.” To cure this violation, the later confirmation of this effect needs to be prevented such that the macroscopic evidence vanishes when additional data are collected. Empirically, this should lead during proceeding observation to a decline of the effect after initial appearance. According to von Lucadou (2015), rather, the effect might unsystematically re-appear on other indicators that were not initially studied. Any effects are thus unsystematically hidden within the additional data acquisition.

As Maier and Dechamps (in press) emphasize, “*the theoretical problem with this approach however is that real null effects documented by replication failures of spurious findings cannot be distinguished from decline effects. The consequence is that with the standard scientific replication approach micro-psychokinesis effects cannot be scientifically studied. Either way, this would mean we should abandon PSI research from science* (for a similar argument see Etzold, 2004) (p. 32).” To solve this dilemma, Maier and Dechamps (in press) adjusted von Lucadou’s model arguing that a violation of the no-signal theorem in quantum physics constitutes a severe violation of the Second Law of Thermodynamics which states that entropy needs to increase over time. The consequence would be that a mentally induced deviation from quantum randomness causes entropy to set in and to counteract this trend. The weaker the quantum effect becomes by this intervention, the quicker the entropic counter-process decreases. This would allow the deviation effect to re-establish itself although with a lowered effect size than initially shown. The authors propose that this interplay continues until the quantum effect has completely vanished. The decline is thus proposed not to be unsystematically drifting toward other indicators but rather follows a systematic pattern of alternations within the same indicator best described as dampened harmonic oscillation of this type:

$$y(t)={ae}^{-\beta t}\cos(\omega t+\varphi )+mt+h$$

With y indicating the effect (e.g., the accumulative z-score) and t representing the additional data collected. The meaning of the parameters for the proposed function can be found in **Table 4**.

**Table 4:** Parameter description of the dampened harmonic oscillation.

Parameter	Meaning
a	Amplitude
β	Decline
ω	Frequency
ϕ	Phase Shift
m	Linear Slope Score
h	Shift along Axis of Ordinates


We propose that the data presented in this study here also follow a similar systematic pattern of decline matching a dampened harmonic oscillation function as suggested by Maier and Dechamps (in press). In the following, we estimated the parameters of the mathematical function shown above for the human data reported here and compared the estimation found with a function derived from simulated data. The simulation was performed with the same experimental design, apparati, and procedures but without human observation. It contains data from 12,571 simulated participants.

### Parameter Estimation for Human Data

Parameter estimation was performed with curve-fitting algorithms provided by the mathematical software tool Wolfram Mathematica Version 11.1.1.0^7^. The mathematical equation mentioned above, reflecting the dampened harmonic oscillation, was provided to the software. The program went through several reiterations, until according to the Maximum Likelihood principle, the group of estimated parameters best fit the empirical data pattern. The approximated function found for the data reported in this study here was:

$$y=-{0,997268e}^{-0,000165877t}\cos(0,0018058t+3,29522)+8,199*10^{-6}t+1,0348$$

**FIGURE 4:**
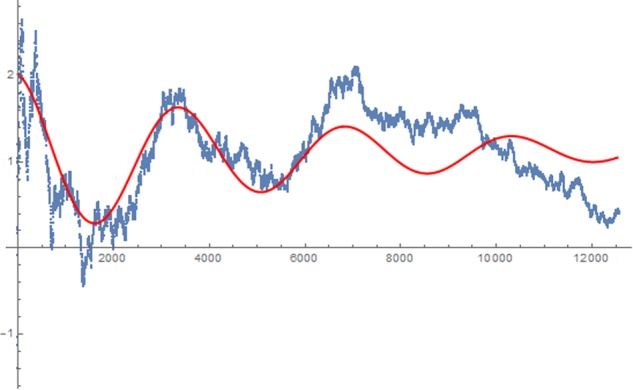
Approximated harmonic oscillation function for the data from all 12,529 participants.

The minimal mean error variance obtained was 0.14 and constitutes the best fit to the data. Any other solution produced a higher error variance. A graphical display of this function together with the empirical effects described as cumulative z-score is displayed in **Figure 4**.

As can be seen, the course of the effect across time approximately follows an alternating pattern, similar to a dampened harmonic oscillation (see red line).

### Parameter Estimation for the Simulation

A simulation was performed to create a control data set for experimental trials without any form of observation. 12,571 data sets were created by running the experimental sessions without any observer being present. Procedure, apparatus, and experimental design were the same as in the human observation condition.

### Results

**FIGURE 5:**
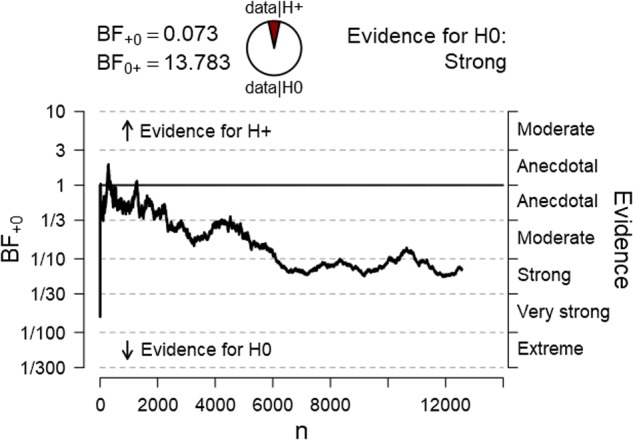
Sequential Bayesian one-sample *t*-test analysis of the percentage of positive pictures obtained by the simulation.

The simulated data were submitted to a Bayesian analysis testing the difference of the average mean score of positive stimuli against chance. A one-sample Bayesian *t*-test was performed revealing a mean score of positive stimuli of *M* = 50.00% (*SD* = 4.99) with a BF_01_ of 13.78, indicating strong evidence for the H_0_. The sequential analysis can be seen in **Figure 5**.

In addition, the same parameter estimation for the dampened harmonic oscillation equation reported above was also performed with the simulated data. The z-transformed accumulated data were submitted to the parameter estimation again with curve-fitting algorithms provided by the mathematical software tool Wolfram Mathematica Version 11.1.1.0^8^. The approximated function found for the simulation was:

y=0,359445⁢ cos⁡(0,000763727t+3,93937)−0,0000966587t+0,85242

**FIGURE 6:**
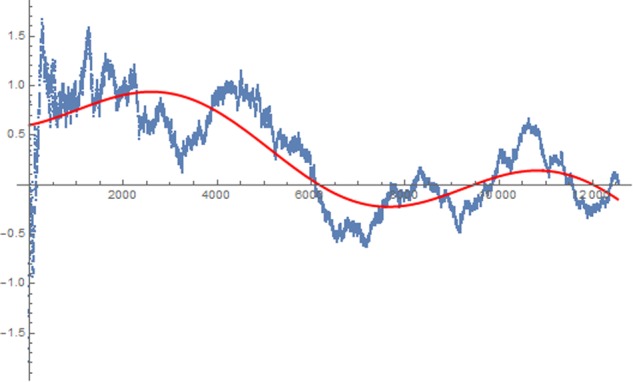
Sequential Bayesian one-sample *t*-test analysis of the percentage of positive pictures obtained by the simulation.

The minimal mean error variance obtained was 0.09. A graphical display of this function together with the cumulative empirical z-scores can be seen in **Figure 6**.

As can be seen from the graph, simulated data can be approximated by a dampened harmonic oscillation function. This is no surprise, since real random effects should initially alternate and with further accumulation asymptotically drift to the zero line. However, in this case, the likelihood for substantial further alternations strongly decreases with additional data generation.

### Comparing Human and Simulated Data

**FIGURE 7:**
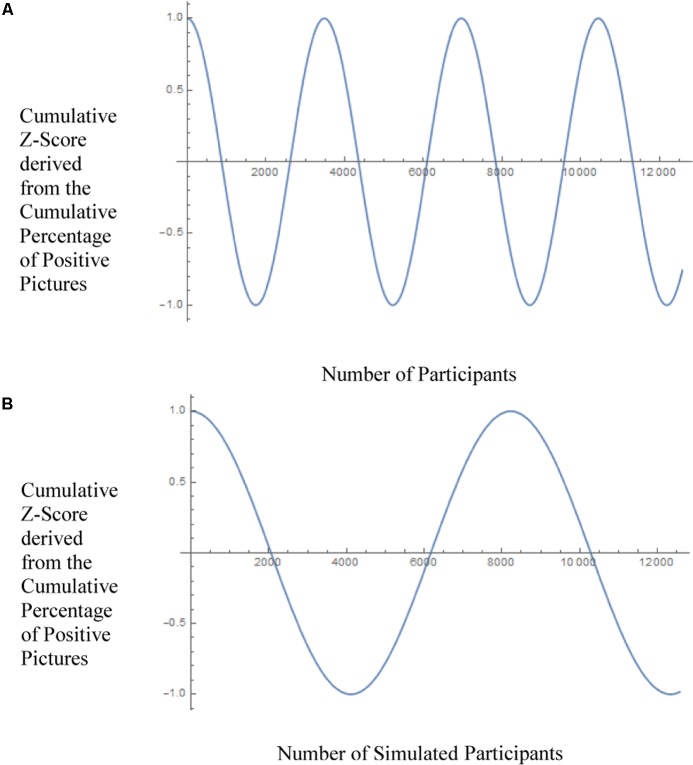
Frequency of the oscillation for real **(A)** and simulated **(B)** participants.

The human and the simulated data should – if the harmonic oscillation assumption is true – differ mainly in the frequency parameter ω. Real effects should produce more pronounced oscillations than artificial data. To explore this, we compared the 95%-confidence intervals for both frequency scores and found indeed that they did not overlap. The frequency score ω obtained with the human data was ω = 0.0018058 with a 95%-confidence interval ranging from [0.00179767; 0.00181392] and the frequency for the simulated data was ω = 0.000763727 with a 95%-confidence interval ranging from [0.000755895; 0.000771559]. Thus, oscillations are much more frequent in the human data than in the simulated control data. This difference is also illustrated in **Figures 7A,B**. This graph only reflects the frequency score when all the other parameters are held constant.

The raw data of the results presented in this manuscript (excluding the personality variables) can be found under: https://open-data.spr.ac.uk/dataset/role-conscious-observation-quantum-randomness-dataset.

### Discussion

In the additional analyses, specific trends in the micro-PK data of our study were investigated. Based on the original explanation for decline effects in PSI, data first presented by von Lucadou (2006, 2015) who assumed a complementary relation between novelty of an effect and its confirmation, Maier and Dechamps (in press) in a re-analysis of their original micro-PK effects proposed that such effects should decline in a systematic way through the interplay between PK-effect and entropy. Specifically, the time course of micro-PK effects should closely match a dampened harmonic oscillation. The pattern of human-related micro-PK oscillations should be different from data obtained without observation. This proposition was tested with the data obtained in the study here. Interestingly, as predicted, the oscillating pattern was different for human as compared to simulated data. The frequency score thus appears to be a good indicator for micro-PK and – assuming that the postulated systematic decline mechanism is true – might be a much better indicator for non-random deviations than the overall mean score obtained in any micro-PK experiment. Future research should focus on systematic decline effects of this nature rather than on normative deviations from chance. Admittedly, at the beginning of this study, this hypothesis and the theoretical background did not exist. It was developed at the end of the data collection from Maier and Dechamps (in press) and applied to the data collected and described here on a *post hoc* basis only. However, we think that it provides a good basis for future research not only on micro-PK but on PSI in general. For now, it is not considered as evidence for micro-PK in the present data. Rather, the goal here was to inform the community about this promising development.

An alternative explanation for this null effect or for the oscillating pattern might also be found in experimenter effects on micro-PK that are specifically tied to the Bayesian approach. The Bayesian sequential analysis demands a continuous monitoring of effect changes due to its stopping rule. The experimenter who is repeatedly watching the updated mean score might develop drifts in his beliefs and by doing so incidentally causes effect changes across time. Varvoglis and Bancel (2015) discuss such experimenter effects in PK research in which the experimenters are considered hidden participants. We are not sure whether this would fully explain the non-existence of the effect or its oscillation, but in future research an “experimentally and theoretically blind” data analyst or an automatic analysis procedure that simply indicates when the stopping criterion is met could be used.

## Conclusion

This study was introduced as a high quality and decisive test for micro-PK. Although several meta-analyses found evidence for micro-PK (Radin and Nelson, 1989, 2003; Bösch et al., 2006), the bulk of the scientific community was not convinced by this form of data aggregation. Rather, they took Carl Sagan’s position arguing that ‘extraordinary claims require extraordinary evidence.’ Brugha et al. (2012) identified a “single high-quality, well-reported study” as one such potential form of extraordinary evidence. We have tried to deliver such a study. The quality features we aimed for were: high power (using a very large sample size), representative sample, high-quality randomization, sophisticated stimuli, objective presentation procedures and high standards on participants’ compliance. All these requirements were met from our team and with the aid of Norstat, a professional polling agency. The results obtained were indeed decisive. Clear and strong evidence for a null effect was found. Thus, micro-PK was not existent in the data. This supports the arguments raised against micro-PK by many skeptics in the field (e.g., Alcock, 2011). It has to be noted that in our study we focused on unconsciously affected intentional states of the observers by using a rather indirect manipulation of our participants’ goals during the picture presentations. The majority of micro-PK studies however have been performed with explicitly induced intentional states. It is unclear how such a manipulation would have affected our participants’ behavior in our study design. Having no such comparison condition is a limitation of our study in terms of generalizability and should be addressed in future micro-PK research.

We would like to emphasize, that the conclusion of “evidence for no effect” is only true when referring to the average mean score of positive stimuli. No deviation from randomness was indeed found with this score. A closer inspection of the temporal change of the effect on the other side revealed some potentially systematic regularity that was not present in the simulated data and can thus hardly be explained by random fluctuations alone. It seemed that the effect in its temporal development across participants behaved like a dampened harmonic oscillation and the amount of oscillations found with human compared to simulated data clearly differed. Maier and Dechamps (in press) explained the existence of such a data pattern through the occurrence of a mechanism called entropy that counteracts the original micro-PK effect. Their mutual interplay most likely produces a dampened harmonic oscillation. If this, admittedly speculative, assumption is true, future PSI research involving quantum RNGs should not focus on significant deviations from chance, but rather should explore oscillating patterns across time and compare these with simulated data. This would be a more fruitful approach than fighting a basic premise in quantum mechanics and it would fit the law of conservation of energy and therefore avoid theoretical paradoxes within science.

In addition, such an oscillating pattern could also be true for standard psychological experiments that involve unconscious processing. Also, for these studies, from a physical point of view, effects are produced effortless and automatic and should therefore also violate the laws of energy conservation and entropy. This should also lead to entropic declines during replication attempts and might also result in an oscillating pattern of effect change. The replication crisis could thus to some extent be also influenced by these mechanisms. We encourage researchers who were working on unconscious processing to analyze their original data and replication attempts accordingly.

Descartes, a famous and well-respected 17th century mathematician and philosopher, was convinced of the existence of micro-PK when he stated that a gambler’s optimistic attitude can bias the outcome of a gambling game toward success. In light of our empirical finding, we would say that he was right only to a certain extent. Indeed, the optimistic gambler might initially achieve higher gains, but then he also has to pay the price of higher losses. Gains and losses during the game will then alternate and approach the chance line at some point. The net earnings will be zero despite the optimistic attitude, but the wins and losses during the game will be more pronounced than in a neutral mood.

## Author Contributions

MM developed theory and hypotheses, designed the study, ran the study, analyzed the data, wrote the first draft; MD designed the study, ran the study, analyzed the data, revised the first draft; MP designed the study, ran the study, revised the first draft.

## Conflict of Interest Statement

The authors declare that the research was conducted in the absence of any commercial or financial relationships that could be construed as a potential conflict of interest. The reviewer JJ and handling Editor declared their shared affiliation.

## Footnotes

1 http://www.norstat.de

2 http://opinion-people.com/dataprotection

3 www.jspsych.org

4 www.shutterstock.com

5 http://www.idquantique.com/random-number-generation/quantis-random-number-generator/

6 The playing times varied slightly between different languages.

7 https://www.wolfram.com/mathematica/

8 http://www.wolfram.com/mathematica/
